# Quantifying Numerical Uncertainty in Background-Oriented Schlieren

**DOI:** 10.21203/rs.3.rs-3349946/v1

**Published:** 2023-09-20

**Authors:** Pranjal Anand, Jiacheng Zhang, Lalit K. Rajendran, Sally P. M. Bane, Pavlos P. Vlachos

**Affiliations:** 1 School of Mechanical Engineering, Purdue University, West Lafayette, IN, USA; 2 School of Aeronautics and Astronautics, Purdue University, West Lafayette, IN, USA

**Keywords:** Background Oriented Schlieren (BOS), uncertainty estimation, Richardson extrapolation

## Abstract

Our study presents and evaluates a method for computing the numerical uncertainty in Background Oriented Schlieren (BOS). We use Richardson extrapolation to assess the uncertainty of numerical integration of density gradients, based on residuals of density results across two grid levels. By integrating this numerical uncertainty with the existing random uncertainty, we obtain the final uncertainty of the density field. We assess the method’s effectiveness using synthetic fields with artificial noise. Our error analysis shows that the sharpness of the density gradient significantly affects bias error and the prediction of numerical uncertainty. The prediction of numerical uncertainty corresponds to variations in bias error, particularly when the noise level and wavelength of the flow field are altered. By accounting for the numerical uncertainty, our method achieves up to 91% accuracy in predicting total uncertainty, as measured against the root-mean-square of the total error. We further demonstrate the utility of our methodology by applying it to experimental BOS images. Our proposed approach offers a more accurate understanding of uncertainty estimation in the BOS technique, with implications for future experiments.

## 1. Introduction

Background Oriented Schlieren (BOS) is an image-based density measurement technique based on the apparent distortion of a target pattern viewed through a medium with varying density (Meier 2002). The pattern distortion is due to the refraction of light rays traversing the medium and can be estimated using cross-correlation, tracking, or optical flow algorithms (Meier 2002). The density gradient can be calculated from the displacement field of the pattern based on the optical layout, and numerical integration of the gradient field provides a spatially resolved estimate of the density (Meier 2002). Due to its simple setup and ease of use, it is currently the most widely used method in quantitative Schlieren diagnostics (Raffel 2015).

**Fig. 1:**
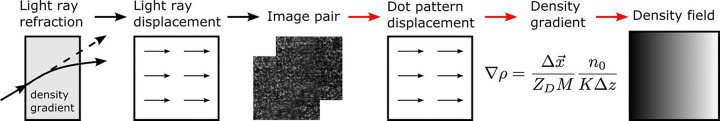
Schematic of the BOS measurement chain. The red arrows indicate the processes that can introduce systematic errors

The BOS measurement chain components can introduce or amplify errors. As shown in Fig. 1 Schematic of the BOS measurement chain. The red arrows indicate the processes that can introduce systematic errors, random error arises from the estimation of the pattern displacement. In contrast, systematic errors can be introduced during the pattern displacement estimation and the calculation and integration of the density gradient. For greater confidence in experimental results, knowledge of the possible systematic error range is important. Therefore, quantifying the uncertainty of BOS measurements is necessary.

A method was recently proposed to estimate the a-posteriori, instantaneous, and spatially resolved density uncertainty for BOS (Rajendran et al. 2020c). The method estimates the uncertainty in the pattern displacement field from cross-correlation using the methods developed for Particle Image Velocimetry (PIV) (Sciacchitano 2019), which is then propagated through the measurement chain to estimate the random uncertainty of the measured density. Since then, methods have been proposed to estimate displacement uncertainties for the dot tracking method (Rajendran et al. 2019, 2020b), and to mitigate the noise sensitivity of the integration procedure by using an uncertainty-based weighted least squares (WLS) approach (Rajendran et al. 2020a). However, the existing uncertainty quantification methods do not account for the systematic uncertainty in the density field, which, based on previous results, could be larger than the random uncertainty, even when the bias error was smaller than the random error in the estimated displacement field (Rajendran et al. 2020c). This systematic error in the measured density is partly due to the discretization errors arising from the numerical integration of density gradients. This work aims to provide uncertainty on this systematic error, referred to as numerical uncertainty.

Numerical uncertainty due to discretization errors is a widely recognized issue in CFD studies and is commonly estimated using Richardson extrapolation (Oberkampf and Roy 2013). This work proposes a method to estimate the numerical uncertainty introduced by the density gradient integration in BOS measurements using Richardson extrapolation. The conventional method for CFD studies is modified to account for the limitations of experimental setups. An approach is also introduced to combine this systematic uncertainty with the random uncertainty to provide an instantaneous, spatially-resolved total uncertainty of the density estimates. The method is validated using synthetic density fields applied to synthetic BOS images and then tested against experimental data.

## 2. Method

### 2.1 Numerical uncertainty estimation for BOS measurements with Richardson extrapolation

In BOS, the density-gradient integration is usually performed using a Poisson solver (Venkatakrishnan and Meier 2004),

(1) $$\rho ={\left(\nabla^{2}\right)}^{-1}(\nabla \cdot \nabla \rho )={\left(G^{T}G\right)}^{-1}\left(G^{T}\nabla \rho \right),$$

where ρ is the density field, ∇ρ is the density gradient field, and G is the gradient operator used for discretizing the derivative, which is also the source of numerical uncertainty considered in this work. Richardson extrapolation estimates the truncation error $\epsilon_{h}$ in the numerical solution $f_{h}$ obtained on a grid with spacing h as:

(2) $$\epsilon_{h}\equiv f_{h}-f=-\frac{f_{h}-f_{rh}}{r^{p}-1}$$

where f is the actual field, $f_{rh}$ is the solution obtained using the same numerical scheme on a coarse grid with spacing rh. Here, r is the grid spacing ratio (set to 2 in the present study) and p represents the order of accuracy. For $\epsilon_{h}$ to be accurate, the solutions $f_{h}$ and $f_{rh}$ need to be in the *asymptotic range,* which requires the error to decrease with mesh refinement at the order of accuracy (Oberkampf and Roy 2013). An *uncertainty bound (U)*, also referred to as the Grid Convergence Index (GCI) (Roache 1994), can be determined from the estimated error as:

(3) $$U=GCI=\frac{F_{S}}{r^{p_{-1}}}\left|f_{h}-f_{rh}\right|$$

where $F_{s}$ is the *Factor of Safety*, a scaling factor that provides an uncertainty bound for a predefined coverage (Roache 1997). The value of $F_{s}$ is chosen empirically to provide a 95% confidence interval for a range of differential equations, discretization procedures, and boundary conditions commonly encountered in CFD problems (Roache 1994). In this study, we propose to use the absolute error from Equation (2) to estimate the standard numerical uncertainty defined as the standard deviation of the numerical error distribution.

As indicated in Equation (2), the order of accuracy p of the integration process is needed to apply Richardson extrapolation for numerical uncertainty estimation. The order of accuracy can be observed from the variation of the errors with two grid spacing levels:

(4) $$p_{e}=\frac{ln\left(\frac{f-f_{rh}}{f-f_{h}}\right)}{lnr}$$

where $p_{e}$ is the order of accuracy based on the errors. However, determining $p_{e}$ is not feasible for experimental BOS because the true solution is unknown in experiments. The order of accuracy can also be observed from the *residuals* between three grid spacing levels:

(5) $$p_{r}=\frac{\text{ln}\left(\frac{f_{r^{2}h}-f_{rh}}{f_{rh}-f_{h}}\right)}{\text{ln}\;r},$$

where $p_{r}$ represents the order of accuracy based on the residuals. For $p_{r}$ to be accurate, the solutions $f_{rh}$ and $f_{r^{2}h}$ on the coarse grids must be smooth and representative of the base solution $f_{h}$ (Oberkampf and Roy 2013), which may not be possible with the resolution of experimental BOS data. Therefore, we propose to use the order of accuracy of the discretization scheme known as the *formal order* $\left(p_{f}\right)$ to approximate the actual order of accuracy of the numerical integration. In the present study, the order of accuracy is assumed to be 2 since the second-order central (SOC) difference scheme was used for the numerical discretization.

The measurement noise in BOS leads to random uncertainty in the obtained density field, and it is desirable to combine the numerical uncertainty with the random uncertainty to provide a *total uncertainty* estimate. Consider the error of a given BOS measurement to be the sum of two random variables, each drawn from the bias and random error distributions of all possible experimental measurements. The variance of the *total* error distribution is the sum of the variances of the bias and random error distributions. By interpreting the numerical uncertainty as the standard deviation of the bias error distribution and the random uncertainty as the standard deviation of the random error distribution, the *standard total* uncertainty defined as the standard deviation of the total error distribution can be expressed as:

(6) $$U_{\mathrm{total}}^{2}=U_{\mathrm{bias}}^{2}+U_{\mathrm{random}}^{2}$$

### 2.2 Synthetic fields

**Fig. 2:**
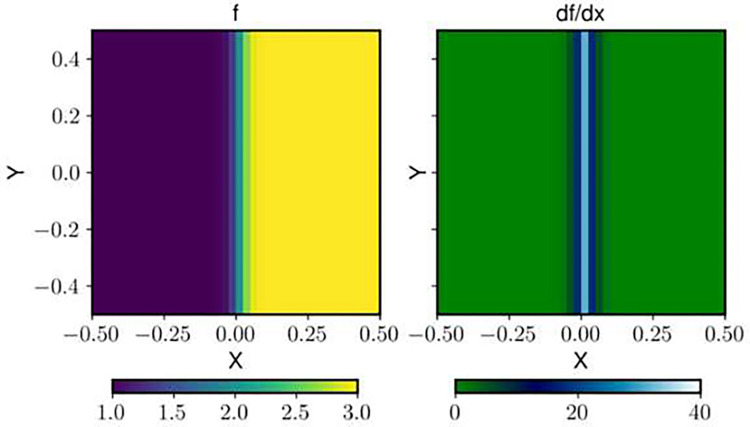
The synthetic field f and its gradient field with a $\lambda_{n}=0.2$

The proposed method is tested on the synthetic fields created from a hyperbolic tangent scalar field described as:

(7) $$f\left(X,Y\right)=2+tanh\left(\frac{2\pi }{\lambda }X\right)\;\frac{df}{dX}\left(X,Y\right)=\frac{2\pi }{\lambda }\left(1-tanh^{2}\left(\frac{2\pi }{\lambda }X\right)\right)$$

where f represents the scalar field and λ represents the wavelength. Such a field is chosen to reflect a real flow field in which BOS might be used, such as that involving a shock. The field $\frac{\partial f}{\partial x}$ is discretely sampled on a regular Cartesian grid with 81 × 81 points and grid spacing h. The range of the domain is −0.5 to 0.5 for both X and Y dimensions. For all plots and results, the wavelength normalized with respect to grid size $\left(\lambda_{n}\right)$ is used. The synthetic scalar and gradient fields with $\lambda_{n}=0.2$ are shown in Fig. 2. The density gradients are integrated using the Poisson solver with the SOC scheme and Dirichlet boundary conditions. The boundary conditions of zero uncertainty and the value of the analytical field are specified only at the left edge. This is done because, in most experiments, boundary condition at only one edge is known (free stream region). The error in the integrated field $\left(f_{h}\right)$ is determined as the deviation from the analytical solution f. To evaluate the method’s robustness in noise and validate the combination framework for the total uncertainty, the gradient fields are corrupted with noise drawn from a zero-mean normal distribution of a prescribed noise level relative to the maximum gradient in the field. Several (1000) realizations of the corrupted field are generated and integrated. The bias uncertainty is estimated for each realization, and the total uncertainty is calculated using Equation (6) with $U_{\mathrm{random}}$ estimated by propagating the uncertainty in the gradient fields to the density fields as in (Rajendran et al. 2020c). The bias error is calculated as the mean value of the error $\left(f_{h}-f\right)$, across all frames for each grid point. The random component is calculated as the standard deviation of the error across all trials, and the total error is the root mean square of the error across all trials.

### 2.3 Experimental BOS images

**Fig. 3:**
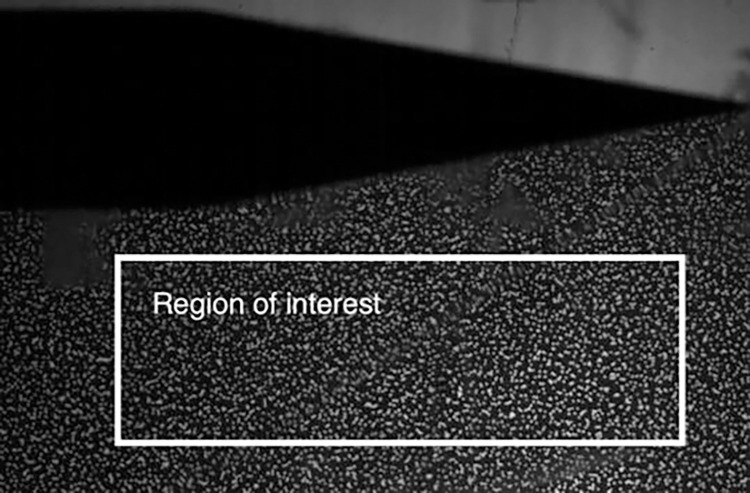
A sample image taken in the experiment with the region of interest indicated (Rajendran et al. 2020c)

The proposed method is demonstrated on measurements from a BOS experiment. The experiment is the supersonic flow over a wedge described in (Rajendran et al. 2020c). The BOS images were taken in a supersonic wind tunnel, of a Mach 2.5 flow over an 11.5◦ wedge with a base of 1 cm and a height of 2.5 cm. One image from the experiment is shown in Fig. 3. The density in the free stream was 0.49 kg/m^3^. The dot pattern consisted of 0.15 mm diameter dots randomly distributed on a transparency with about 25 dots per 32 × 32 pixel window. Further details of the experimental setup are provided in (Rajendran et al. 2020c). The images were processed using a multi-pass window deformation approach for two passes with identical window sizes and window resolution of 32×32 px^2^. The window overlap was set to 0%. The intermediate passes were smoothed but not validated to avoid the vectors in the shock region being identified as outliers (sharp changes are characteristic of this flow). The processing and uncertainty calculation was performed using an open-source code PRANA (https://github.com/aether-lab/prana/). Displacement uncertainty calculation from image matching is used (Rajendran et al. 2020c). After processing, the vectors are corrected to account for camera vibrations by subtracting the mean displacement in the free stream region (which is not supposed to show any displacement vectors) from all the displacement vectors.

The depth-averaged density gradient field ∇ρ is calculated from the displacement field using the BOS optical model as:

(9) $$\nabla \rho =\frac{\Delta \vec{x}}{Z_{D}M}\frac{n_{0}}{K\Delta z}$$

where $\Delta \vec{x}$ is the displacement, M is the magnification of the dot pattern, $Z_{D}$ is the distance between the dot pattern and the mid-point of the density gradient field, $n_{0}$ is the ambient refractive index, K is the Gladstone-Dale constant (= 0.225 × 10^−3^ m^3^/kg for air) (Raffel 2015). The gradients are spatially integrated using the Poisson solver with a Dirichlet boundary condition of 0.49 kg/m^3^ at the right edge (free stream) to obtain the projected density field.

This is done for all 5000 images of the second pass. The displacement uncertainty is propagated similarly with a Dirichlet boundary condition of 0.0 kg/m^3^ at the right edge. For each grid point, the ensemble average of numerical uncertainty is calculated, and the RMS of the random uncertainty is calculated, across all frames. Finally, the total uncertainty is calculated from Equation (6).

## 3. Results

### 3.1 Analysis with synthetic fields

To determine the order of accuracy of the density gradient integration, $p_{e}$ and $p_{r}$ were calculated using Equations (4) and (5), respectively, and compared to the formal order $\left(p_{f}\right)$ from the density fields with $\lambda_{n}$ varying from 0.1 to 1.0. As shown in Table 1, the formal order $\left(p_{f}\right)$ matched the error-based order $\left(p_{e}\right)$ for all cases, while the residual-based order $\left(p_{r}\right)$ began to deviate from the other two as the wavelength decreased.

**Fig. 4:**
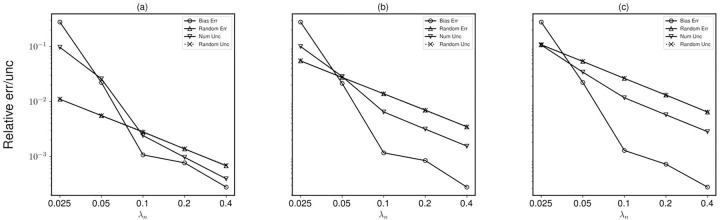
The comparison between the RMS of the bias and random errors with the numerical and random uncertainty RMS for the synthetic fields for all $\lambda_{n}$ for noise levels (a) 1%, (b) 5% and (c) 10%

Fig. 4 shows the trend of the normalized (with respect to the maximum value of density) bias and random errors and uncertainties with changing wavelength and noise levels. For a $\lambda_{n}$ of 0.1, the shock is captured by 3 pixels, and for $\lambda_{n}=0.4$, the gradient is captured by 9 pixels (5). The relative bias error at $\lambda_{n}=0.1$ is 95% lower than that at $\lambda_{n}=0.05$. The random error and random uncertainty increase with both sharpness of the gradient and increasing noise level. The numerical uncertainty has a direct relation with noise but an inverse relationship with wavelength. 5 shows how a sharper gradient (lower $\lambda_{n}$) affects the bias error and numerical uncertainty prediction.

**Fig. 6:**
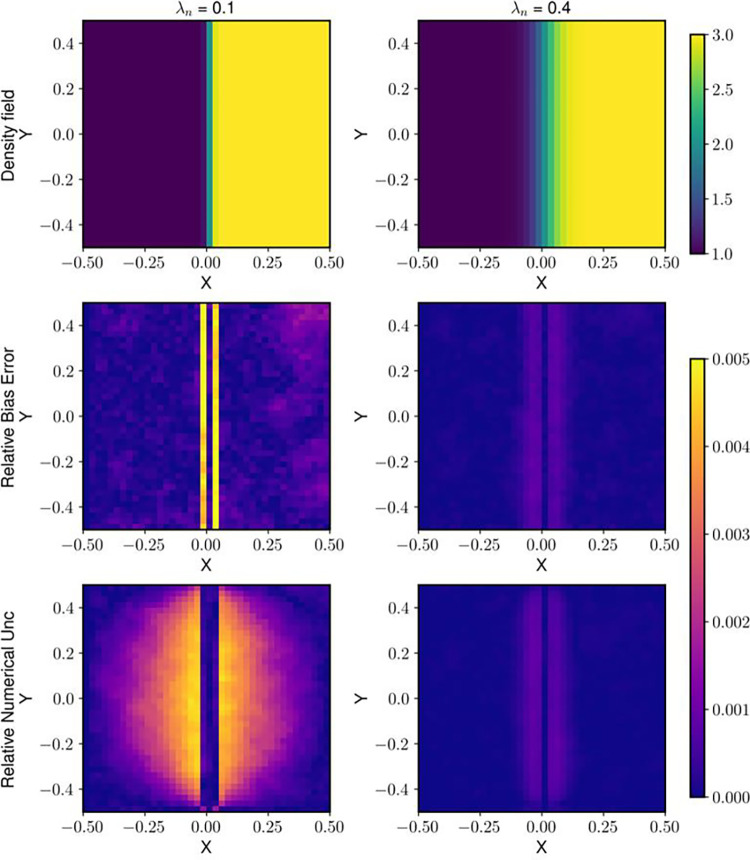
The density fields and spatial distributions of bias error and numerical uncertainty of the syathetic field a $\lambda_{n}=0.1$ and $\lambda_{n}=0.4$ at 5% noise

With a sharper gradient of $\lambda_{n}=0.1$, the root mean square (RMS) of the total uncertainty is within 21% of the RMS of total error for 1% noise, and within 9% of the total error for 10% noise (Fig. 6). For $\lambda_{n}=0.4$, (less sharp gradient), the total uncertainty is within 8% for both the lowest and highest noise levels. Another key observation is the difference in numerical uncertainty as noise level increases for the same wavelength. For $\lambda_{n}=0.025$, the relative increase in numerical uncertainty from 1% to 10% noise level is just 3% while for $\lambda_{n}=0.1$ the increase is over 4 times that of the value at 1% noise level. For sharper gradients, as the bias error and numerical uncertainty are already quite high, their increase is relatively smaller.

### 3.2 Analysis with experimental BOS images

**Fig. 7:**
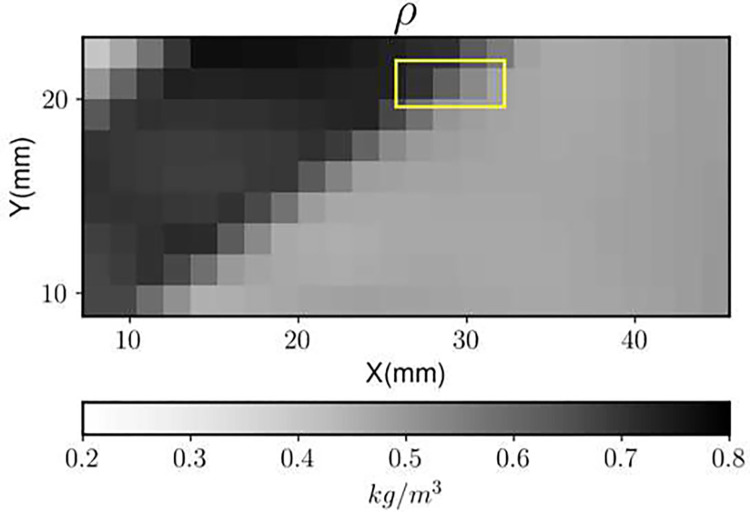
The ensemble-averaged density field, with the shock indicated

**Fig. 8:**
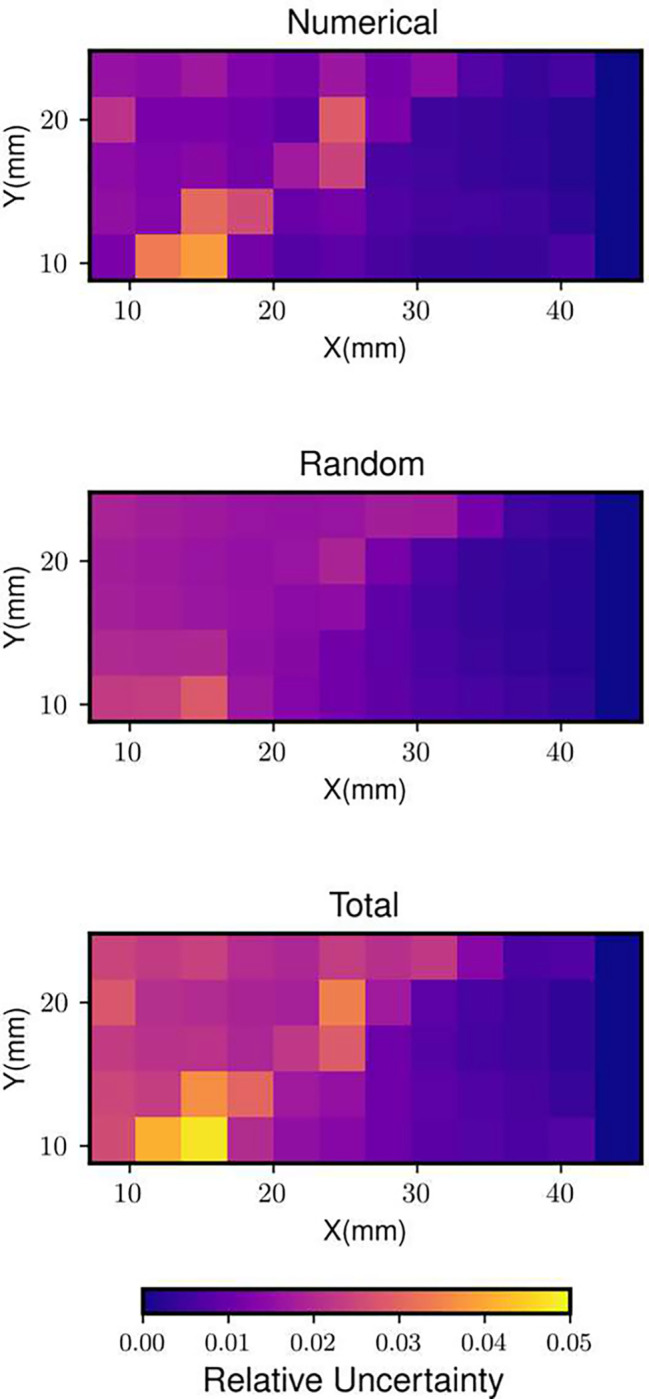
The spatial distributions of the uncertainty and its components from the experimental BOS images

**Fig. 9:**
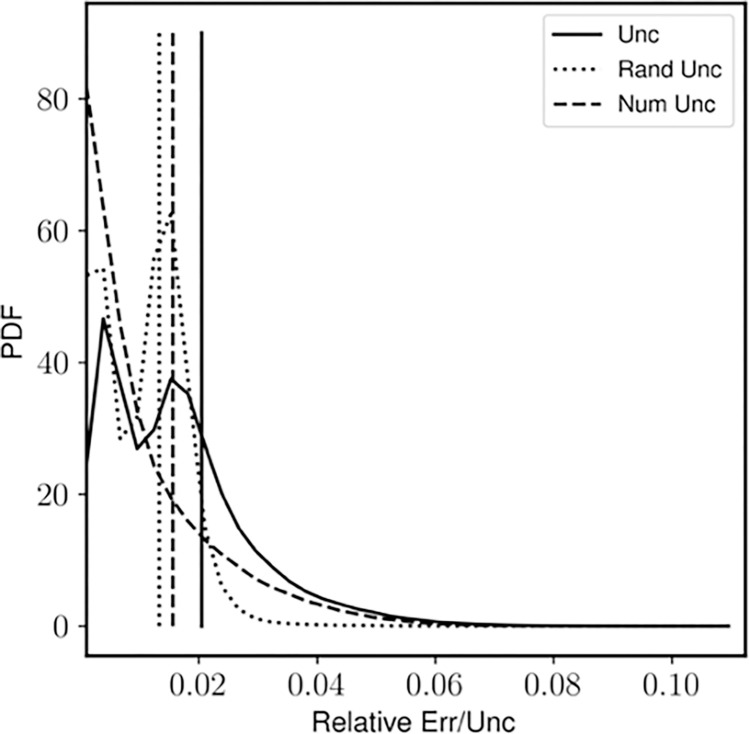
The statistical distributions of the total error and uncertainty from the experimental BOS images. The straight lines are the RMS values of the respective quantities

The density field obtained from the experimental BOS images and the numerical uncertainty results (normalized with respect to the maximum value in the density field) are shown in Fig. 7 and Fig. 8, respectively. The shock is captured across three pixels as can be seen from the density field. The resulting uncertainty distribution is bimodal. The peaks correspond to the random uncertainty before and after the shock respectively (Rajendran et al. 2020c). In this case the RMS of random uncertainty is greater than that of numerical uncertainty (Fig. 9). This may be because Image Matching is used for calculating the displacement field uncertainty, which reports a higher random uncertainty compared to other methods (Rajendran et al. 2020c) for this experiment.

## 4. Discussion and conclusions

In this study, we proposed and applied a method to estimate the numerical uncertainty for BOS density measurement. Based on the differences between the density results obtained on multiple grid levels, the numerical uncertainty is estimated using Richardson extrapolation. Compared to the framework of applying Richardson extrapolation to CFD simulations, several adaptations are made considering the limitations of experimental BOS measurements. First, the formal order of the numerical discretization is used as the order of accuracy of the numerical integration for performing Richardson extrapolation. Previous CFD studies showed that the observed order of accuracy can be lower than the formal order for the simulations of the flow fields governed by hyperbolic equations with strong discontinuities (Oberkampf and Roy 2013), thus the observed order of accuracy using Equation (4) or (5) are normally preferred. However, determining the order of accuracy from solution errors is not feasible as the true solution is normally unavailable for experimental BOS, and observing the order of accuracy from the solution residuals can fail due to the limited spatial resolution of the experimental data. Since the Poisson equation solved for the density gradient integration is elliptic, the order reduction concern may not apply to BOS. As shown in Table 1, the formal order matched with the order of accuracy observed from the solution errors, while the order of accuracy observed from the solution residuals failed for low-wavelength fields due to the insufficient spatial resolutions of the coarser grid levels, suggesting that the formal order is a suitable choice for the BOS numerical uncertainty estimation. Moreover, instead of determining the numerical uncertainty based on the empirically selected factor of safety (Fs), we propose to use absolute error obtained from Equation (2) to estimate the standard numerical uncertainty, which is validated by the comparison between the bias error with the numerical uncertainty from the synthetic fields as shown in Fig. 4. In addition, we proposed a framework to obtain the total uncertainty of the density measurement by combining the numerical uncertainty with the random uncertainty which is not seen in CFD simulations.

The analysis with the experimental BOS images and synthetic field test cases indicated that the contribution of the numerical uncertainty to the systematic error of the density field varies with the sharpness of the density gradient. Fig. 6 shows that the numerical uncertainty increases with the distance from the right boundary, for which the uncertainty was set to be 0. Additionally, the numerical uncertainty in the regions of the shock is elevated, which is expected due to the sharp density gradient present which cannot be well resolved.

There are several limitations of the proposed numerical uncertainty prediction method. First, the proposed method can be affected by the measurement noise, as seen from the synthetic field results in Fig. 4, where greater noise leads to the overprediction of bias errors. Additionally, shocks and discontinuities, like that present in the experimental images, invalidate the Taylor series approximation and hence also the Richardson Extrapolation (Roache 1994), leading to potential inaccuracy in estimating numerical uncertainty. It is also worth mentioning that the numerical integration is not the only source of bias error in the BOS measurement chain. Other sources, for instance, the error due to the simplified optical model for evaluating the density gradient from light ray displacement, will be investigated in future work.

## Figures and Tables

**Fig. 5:**
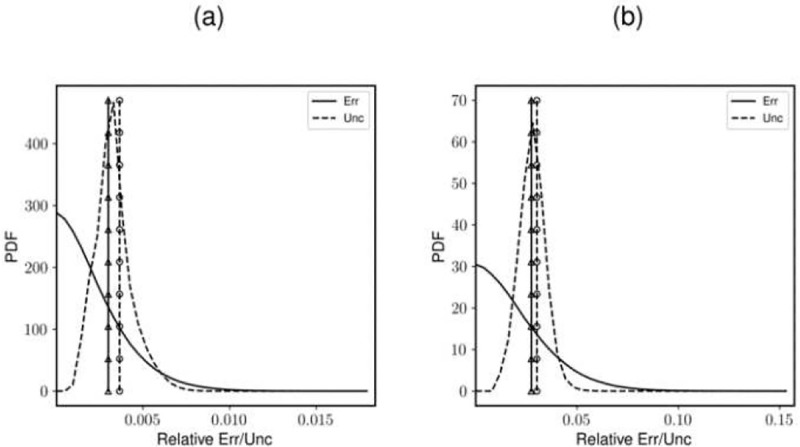
The statistical distributions of the total error and uncertainty from the syathetic fields with a $\lambda_{n}$ of 0.1 and (a) 1% noise and (b) 10% noise

**Table 1:** The order of accuracy determined using different methods for the numerical integration of the density gradient with a SOC scheme

wavelength λ	0.1	0.5	1.0

Formal order $\left(p_{f}\right)$	2	2	2
Error-based order $\left(p_{e}\right)$	1.51	2	2
Residual-based order $\left(p_{r}\right)$	3.28	1.83	1.82

## Data Availability

The supersonic flow over wedge dataset used for the demonstration can be found on Purdue University Research Repositories-https://purr.purdue.edu/publications/3758/.

## References

[R1] Meier G (2002) Computerized background-oriented schlieren. Exp Fluids 33:181–187. 10.1007/s00348-002-0450-7

[R2] Oberkampf WL, Roy CJ (2013) Solution verification. In: Verification and Validation in Scientific Computing. pp 250–285

[R3] Raffel M (2015) Background-oriented schlieren (BOS) techniques. Experiments in Fluids 56:1–17. 10.1007/s00348-015-1927-5

[R4] Rajendran L, Zhang J, Bane S, Vlachos P (2020a) Uncertainty-based weighted least squares density integration for background-oriented schlieren. Experiments in Fluids 61:1–12. 10.1007/s00348-020-03071-w

[R5] Rajendran LK, Bane SPM, Vlachos PP (2019) Dot tracking methodology for background-oriented schlieren (BOS). Experiments in Fluids 60:1–13. 10.1007/s00348-019-2793-3

[R6] Rajendran LK, Bane SPM, Vlachos PP (2020b) Uncertainty amplification due to density/refractive index gradients in background-oriented schlieren experiments. Experiments in Fluids 61:1–16. 10.1007/s00348-020-02978-8

[R7] Rajendran LK, Zhang J, Bhattacharya S, (2020c) Uncertainty quantification in density estimation from background-oriented Schlieren measurements. Measurement Science and Technology 31:. 10.1088/1361-6501/ab60c8

[R8] Roache PJ (1994) Perspective: A method for uniform reporting of grid refinement studies. Journal of Fluids Engineering, Transactions of the ASME 116:405–413. 10.1115/1.2910291

[R9] Roache PJ (1997) Quantification of uncertainty in computational fluid dynamics. Annual Review of Fluid Mechanics 29:123–160. 10.1146/annurev.fluid.29.1.123

[R10] Sciacchitano A (2019) Uncertainty quantification in particle image velocimetry. Measurement Science and Technology 30:. 10.1088/1361-6501/ab1db8

[R11] Venkatakrishnan L, Meier GEA (2004) Density measurements using the Background Oriented Schlieren technique. Experiments in Fluids 37:237–247. 10.1007/s00348-004-0807-1

